# A Nonequilibrium Model for Particle Networking/Jamming and Time-Dependent Dynamic Rheology of Filled Polymers

**DOI:** 10.3390/polym12010190

**Published:** 2020-01-10

**Authors:** Christopher G. Robertson, Sankar Raman Vaikuntam, Gert Heinrich

**Affiliations:** 1 Endurica LLC, Findlay, OH 45840, USA; 2 Leibniz-Institut für Polymerforschung Dresden e.V., 01069 Dresden, Germany; sankarraman.vaikuntam@gmail.com (S.R.V.); gheinrich@ipfdd.de (G.H.); 3 Institut für Textilmaschinen und Textile Hochleistungswerkstofftechnik, Technische Universität Dresden, 01069 Dresden, Germany

**Keywords:** polymer nanocomposites, filled rubber, particle network, filler flocculation, fictive strain, structural relaxation, Tool–Narayanaswamy–Moynihan model, jamming

## Abstract

We describe an approach for modeling the filler network formation kinetics of particle-reinforced rubbery polymers—commonly called filler flocculation—that was developed by employing parallels between deformation effects in jammed particle systems and the influence of temperature on glass-forming materials. Experimental dynamic viscosity results were obtained concerning the strain-induced particle network breakdown and subsequent time-dependent reformation behavior for uncross-linked elastomers reinforced with carbon black and silica nanoparticles. Using a relaxation time function that depends on both actual dynamic strain amplitude and fictive (structural) strain, the model effectively represented the experimental data for three different levels of dynamic strain down-jump with a single set of parameters. This fictive strain model for filler networking is analogous to the established Tool–Narayanaswamy–Moynihan model for structural relaxation (physical aging) of nonequilibrium glasses. Compared to carbon black, precipitated silica particles without silane surface modification exhibited a greater overall extent of filler networking and showed more self-limiting behavior in terms of network formation kinetics in filled ethylene-propylene-diene rubber (EPDM). The EPDM compounds with silica or carbon black filler were stable during the dynamic shearing and recovery experiments at 160 °C, whereas irreversible dynamic modulus increases were noted when the polymer matrix was styrene-butadiene rubber (SBR), presumably due to branching/cross-linking of SBR in the rheometer. Care must be taken when measuring and interpreting the time-dependent filler networking in unsaturated elastomers at high temperatures.

## 1. Introduction

There is considerable academic and industrial interest in the rheology/viscoelasticity of polymer systems due the influence on both the processability in manufacturing and the final product performance. One important example is in the field of automobile tire technology where the dynamic mechanical behavior of the tread compound is closely connected to—and predictive of—the fuel economy, traction, and handling/cornering performance characteristics of a tire [1,2]. For general background information about polymer viscoelasticity, several excellent books on this technical area are suggested [3,4,5,6].

Temperature and frequency are the main experimental variables in dynamic mechanical characterization of polymer materials. Strain amplitude effects are also of critical importance in polymer composites reinforced by particles. First documented by Dillon, Prettyman, and Hall in 1944 [7], the Payne effect [8,9,10] is a well-known viscoelastic phenomenon in particle-filled elastomers that is characterized by a significant reduction in dynamic storage modulus and the appearance of a peak in loss tangent (tanδ) as the oscillatory strain amplitude is increased. This hysteretic softening occurs at small dynamic strains, with most of the storage modulus reduction taking place in the range from 0.1 to 10% strain. Unfilled elastomers do not typically exhibit strain-dependent viscoelastic response until much higher strain amplitudes (>50%). The key features of the Payne effect are described in a chapter by Heinrich and Klüppel [11] and in a brief review [12]. The tire industry is particularly interested in understanding the Payne effect and developing materials technologies to reduce its magnitude because of the impact of this viscoelastic behavior on fuel economy. The global oil consumption each day for ground vehicles is over 50 million barrels of oil [13]. About 10% of the fuel used by an automobile is consumed to overcome the rolling resistance of tires, and at least half of that rolling resistance is from the Payne effect of the rubber compounds within the tires. Therefore, more than 2.5 million barrels of oil are wasted each day around the world due to the Payne effect of tire compounds.

**Figure 1:**
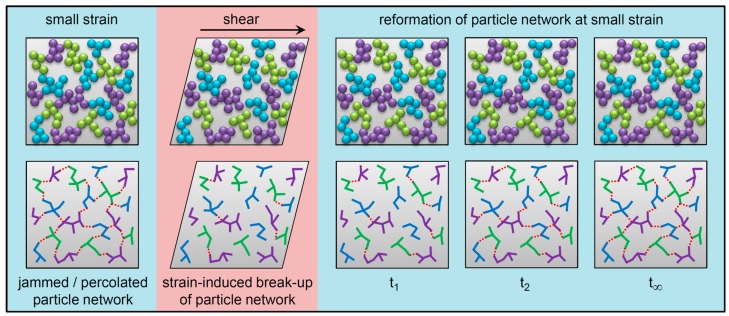
Illustration of the effect of shear strain on the particle network in a filled elastomer and the subsequent time-dependent reformation process. The different colored aggregates in the upper diagrams represent fused primary particles, and these aggregates are the smallest dispersible units of nano-structured fillers such as carbon black and precipitated silica. The lower diagrams illustrate the connectivity of the filler network, with solid lines showing fused connections of primary particles within aggregates and dashed lines showing aggregate–aggregate (filler–filler) contacts.

The presence of a strain-sensitive filler network composed of percolated particle–particle contacts is responsible for the majority of the Payne effect [11,14,15,16,17], although polymer dynamics at the polymer–filler interfaces also have a secondary contribution [15,18,19,20]. Once the shearing of an uncross-linked-filled elastomer compound is ceased, the filler network strengthens with time which leads to a larger Payne effect. This is commonly referred to as filler flocculation, and it has been widely studied across the past two decades [21,22,23,24,25,26,27,28,29,30,31,32,33,34,35,36]. Once a filler network is formed, it can be broken by deforming the material above about 20% strain, and the filler network will reform with time after the strain is removed or reduced. This is schematically illustrated in Figure 1 for nano-structured fillers such as precipitated silica and carbon black made up of fused aggregates of nanometer-scale primary particles. Only very small movements of aggregates are needed to break the connectivity of the filler network, and reformation of the network takes place across correspondingly small distances. Therefore, the term flocculation is somewhat of a misnomer for this filler network build-up phenomenon. 

The physics of particle systems have similarities to glassy behavior, but with deformation (stress, strain, vibration) driving the response instead of temperature as the key parameter. The noted similarities between temperature effects in glass-forming materials and influence of deformation on particle systems led to the creation of jamming phase diagrams [37,38]. These ideas have been extended to particle-filled rubber [39,40]. Phenomenological modeling of time-dependent properties during relaxation in the nonequilibrium glassy state is well established, which provides the opportunity to borrow those concepts for modeling the filler networking/jamming process in filled polymers by substituting deformation for temperature. This present study will show dynamic rheological results that reveal filler networking/flocculation in model-filled rubber formulations, and glassy modeling approaches will be adapted to fit the time-dependent data and give new insights into the behavior. Given the known importance of relative surface energies for polymer and particles to the filler flocculation process [33,34,35,41,42], our investigation included two different elastomers, ethylene-propylene-diene rubber (EPDM) and styrene-butadiene rubber (SBR), that are reinforced with two different fillers: precipitated silica and carbon black (CB). 

## 2. Materials and Methods

The polymers included in this investigation were EPDM (Buna EP G 3440) and solution SBR (VSL 2525 0 m) from Arlanxeo Deutschland GmbH (Dormagen, Germany). The precipitated silica (Ultrasil VN3 grade) from Evonik Industries AG (Essen, Germany) and carbon black (N115 grade) from Orion Engineered Carbons GmbH (Cologne, Germany) have nitrogen surface areas of 180 and 137 m^2^/g, respectively, as reported by the suppliers. The antioxidant used was rubber grade 6PPD (N-(1,3-dimethylbutyl)-N′-phenyl-p-phenylenediamine). 

The simple model rubber formulations in Table 1 were mixed using a Haake Rheomix 600P (Thermo Fisher Scientific GmbH in Karlsruhe, Germany). This mixer has a chamber volume of 80 cc, which was filled 70% with compound during mixing. Using a starting temperature of 110 °C and rotor speed of 60 rpm, the compounds were mixed for 8 min to a final temperature of 140 to 150 °C. The compounds were then milled for 2 min using a two-roll mill (Polymix-110L; Servitec Maschinenservice GmbH in Wustermark, Germany) at 50 °C with a friction ratio of 1:1.2. Densities for the raw materials were used to determine values of filler volume fraction (φ), which was nearly constant at 0.15 to 0.17 for the four compounds (Table 1).

Oscillatory shear rheology measurements were conducted using a Rubber Process Analyzer (RPA) made by Scarabaeus GmbH (Wetzlar, Germany; model SIS-V50) that uses a serrated biconical die geometry. All RPA testing was performed at a temperature of 160 °C and frequency (f) of 1.67 Hz (angular frequency (ω) = 10.5 rad/s). The dynamic strain amplitude (*γ*) was varied in a time sequence: (1) 5 min at *γ* = 0.25 to break up the filler network; (2) 120 min at the flocculation *γ* value; and (3) 10 min each at sequentially increasing strains up to and including *γ* = 0.25. A fresh rubber compound specimen was used for each RPA testing sequence, and the volume of compound used for testing was approximately 5 cm^3^. We report results for shear storage modulus (G’) and the magnitude of complex viscosity, |η*|, which is commonly called dynamic viscosity and represented without the brackets, η*.

## 3. Results and Discussion

This study considered simple SBR and EPDM formulations containing only polymer, filler, and an antioxidant (Table 1). The filler volume fraction was nearly constant (φ = 0.15 to 0.17) for these materials which did not contain any cross-linking additives. The CB and silica filler grades used have specific surface areas of 137 m^2^/g and 180 m^2^/g. Although these are nano-structured fillers (see Figure 1), we can get an idea of the size of these filler materials by converting the specific surface areas to values of equivalent spherical particle diameter (d). The surface to volume ratio of a sphere is 6/d which leads to d = 17 nm for silica and d = 24 nm for CB using densities for silica and carbon black of 2.0 and 1.8 g/cm^3^, respectively. The filled elastomers investigated here are clearly polymer nanocomposites. 

**Figure 2:**
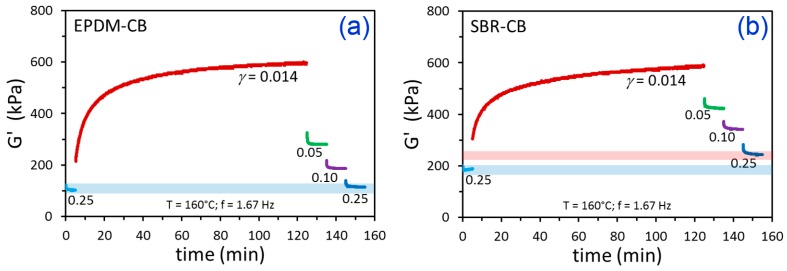
Time-dependent G’ results for the indicated strain amplitude history for EPDM-CB (**a**) and SBR-CB (**b**). The blue bar in (**a**) represents the similar starting and ending values of G’ at *γ* = 0.25. In (**b**), the blue bar is the starting G’ at *γ* = 0.25 and the pink bar is the final G’ at *γ* = 0.25, which is higher.

**Figure 3:**
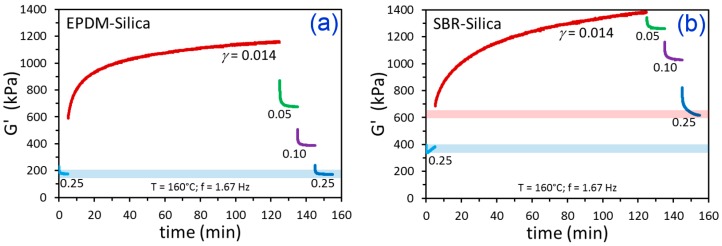
Time-dependent G’ results from the indicated strain amplitude history for EPDM-Silica (**a**) and SBR-Silica (**b**). The blue bar in (**a**) represents the similar starting and ending values of G’ at *γ* = 0.25. In (**b**), the blue bar is the starting G’ at *γ* = 0.25 and the pink bar is the final G’ at *γ* = 0.25, which is higher.

Oscillatory shear rheology was used to study filler networking kinetics for CB-filled and silica-filled SBR and EPDM. It is common to use temperatures in the 150 to 170 °C range for studying filler flocculation in rubber, because this is the typical range where curing (vulcanization) of rubber compounds takes place commercially. Most of the filler networking occurs during the early stages of curing, before the polymer chains become cross-linked [30]. We employed a temperature of 160 °C for all of our measurements. The testing protocol involved: (1) breaking up the filler network at a strain amplitude (*γ*) of 0.25 (25%); (2) down-jump to the flocculation *γ* where changes in rheological properties were monitored for 120 min; and (3) sequential break-up of the filler network at various increasing strains, each applied for 10 min, up to the final *γ* of 0.25. So, the testing series started and ended at the same conditions. The storage modulus (G’) results for this testing series are shown in Figure 2 for the CB-filled polymers using a flocculation *γ* of 0.014 and subsequent filler network break-up *γ* values of 0.05, 0.10, and 0.25, and the results for the silica filler are given in Figure 3 using the same conditions. For both types of filled EPDM, it is evident that the starting and ending G’ at *γ* = 0.25 were essentially the same. When the polymer matrix was SBR, however, irreversible increases in G’ were noted, presumably due to branching/cross-linking of SBR in the rheometer. Cross-linking of unsaturated elastomers without vulcanization agents at high temperatures is a known occurrence [43,44,45]. This grade of SBR has a structure with 75 wt.% butadiene (unsaturated; C=C double bond in every polymer repeat unit from butadiene), whereas the EPDM has a nearly fully saturated structure except for the sparse double bonds from the 4.1 wt.% ethylidene norbornene comonomer. For filler flocculation studies, we recommend a testing sequence like the one utilized here and/or a time sweep on the unfilled polymer to ensure that the material is stable, such that any kinetic model fitting—and parameter interpretation therefrom—can be considered valid and meaningful. The remainder of the experiments to be discussed and the phenomenological model fitting will only involve the stable EPDM-CB and EPDM-silica materials.

For studying filler flocculation, the time dependence of G’ is often used, and examples from our testing were just presented in Figure 2 and Figure 3. However, for our detailed analysis and model fitting, we use the magnitude of the complex viscosity, |η*|, which is equal to |G*|/ω where |G*| is the magnitude of the complex modulus and ω is the angular frequency (ω = 2πf). We represent this simply as η* (without the brackets) and refer to this quantity as dynamic viscosity. We study η* for two reasons: (1) it is directly determined from the experimental stress and strain amplitudes without having to separate the dynamic shear response into storage modulus and loss modulus and accordingly prove the validity of linear-nonlinear dichotomy for our filled rubber compounds at the testing conditions [20,30,46]; and (2) viscosity is a typical property used to describe glass-forming materials, to which we will draw parallels later. 

**Figure 4:**
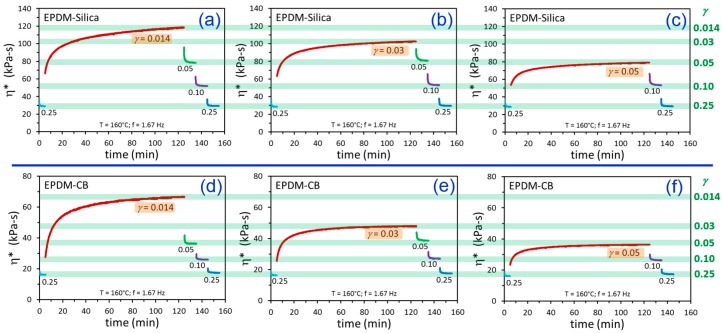
Time-dependent dynamic viscosity results for EPDM-Silica (**a**–**c**) and EPDM-CB (**d**–**f**) from testing using the indicated strain amplitude histories. The green bars represent the apparent equilibrium η* values at the various values of *γ*. Note the different y-axis scalings for EPDM-Silica (**a**–**c**) and EPDM-CB (**d**–**f**).

The build-up of dynamic viscosity after a down-jump from equilibrium at *γ* = 0.25 was studied at three different networking/flocculation *γ* values of 0.014, 0.03, and 0.05. The data in Figure 4 indicate significantly more extensive filler networking for silica relative to CB in the EPDM matrix. This is expected since the bare silica without silane surface modification is polar (surface covered with -OH groups) and consequently has much stronger filler–filler interactions as flocculation driving forces compared to carbon black. Another observation for both materials is that the degree of viscosity growth increased as the flocculation strain was reduced.

**Figure 5:**
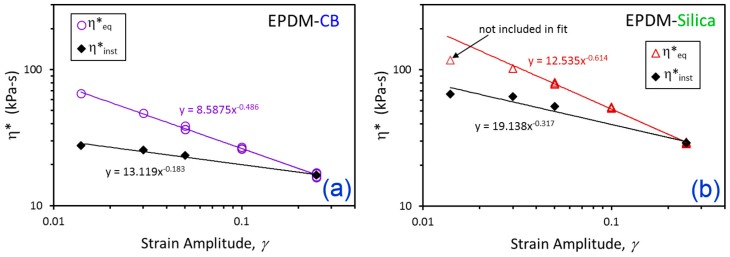
*γ*-dependent values of η*_eq_ and η*_inst_ (symbols) and power law fits (solid lines) for EPDM-CB (**a**) and EPDM-Silica (**b**).

A key observation from Figure 4 is that for each polymer–filler system, there appears to be an equilibrium dynamic viscosity, η*_eq_, at each value of *γ* that is independent of the prior strain history path. This is highlighted by the green bars in Figure 4, and the *γ*-dependence of η*_eq_ displays a power law behavior for both EPDM-CB and EPDM-Silica (Figure 5).

(1) $${\;\eta *}_{\mathrm{eq}}=c\;\gamma^{-\alpha }$$

The power law parameters are summarized in Table 2. Switching the independent and dependent variables produces an expression that will be used later to define the time dependence of fictive strain, *γ_f_*, in terms of the dynamic viscosity evolution with time:

(2) $$\gamma_{f}\left(t\right)=c^{1/\alpha }\;{\left[\eta^{*}\left(t\right)\right]}^{-1/\alpha }$$

Another noted aspect of the general behavior in Figure 4 is that there seems to be a discontinuity in η* when the strain is suddenly reduced from equilibrium at *γ* = 0.25 to the flocculation *γ*. The time between the last datapoint at *γ* = 0.25 and the first datapoint at the flocculation strain is 6 s, so it is possible that the noted viscosity jump in each case is a consequence of this time gap (i.e., missing data). Interestingly, however, this “instantaneous” dynamic viscosity, η*_inst_, also shows a power law dependence with respect to *γ*, with parameters reported in Table 2.

(3) $${\eta *}_{\mathrm{inst}}=b\;\gamma^{-\theta }$$

The apparent viscosity jumps may reveal some real physics of the flocculation process, namely that it is composed of an instantaneous part and a time-dependent part. 

The nonequilibrium behavior of glasses is modeled using actual temperature (*T*) and fictive temperature (*T_f_*) [47,48,49,50], and the analogous concept of fictive strain was recently introduced for filled rubber [32]. Given the similarities between the influence of deformation on jammed particle systems and temperature effects on glass-forming materials, we adapt the Tool–Narayanaswamy–Moynihan (TNM) model [47,48,51,52] that is used to represent the structural relaxation (physical aging) process in the nonequilibrium glassy state. Substituting *γ* and *γ_f_* for *T* and *T_f_* in the TNM approach leads to the fictive strain model:

(4) $$\eta^{*}\left(t\right)\;=\;{\eta *}_{0}+(\;{\eta *}_{\infty }-\;{\eta *}_{0})\{1-\exp\left[-{\left(\overset{t}{\underset{0}{\int }}\frac{\mathrm{dt}}{\tau \left(\gamma ,\gamma_{f}\left(t\right)\right)}\right)}^{\beta }\right]\}$$

(5) $$\;\tau =A\;exp\left[\frac{a}{\gamma }+\frac{s}{\gamma_{f}\left(t\right)}\right]\;$$

**Figure 6:**
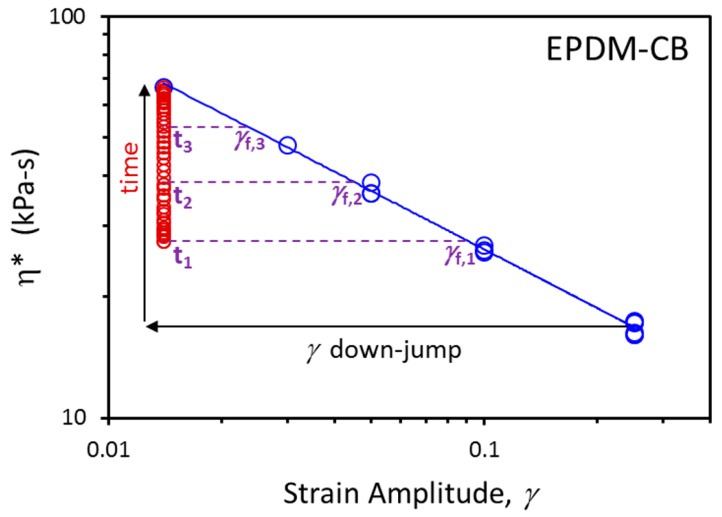
Illustration of fictive strain determination during time-dependent η* build-up (red symbols) at *γ* = 0.014 after strain down-jump from *γ* = 0.25 for EPDM-CB. The blue symbols are η*_eq_ vs. *γ* along with the related power law fit (solid line).

It should be noted that the functional form of Equation (5) is different than a preliminary expression proposed earlier for the relaxation time, *τ* [32]. At first glance, the expression in Equation (4) looks like a typical stretched exponential growth function, with stretching exponent *β*. However, the unique part is the relaxation time function, which depends on actual strain through the parameter *a* and fictive/structural strain through the parameter *s* (Equation (5)). The fictive strain decreases toward the actual strain during flocculation, thereby imparting a time-dependent increasing nature to τ as filler networking progresses. The *γ_f_*(*t*) is assigned from the measured η*(t) using Equation (2), and a visual example of the connection between the time dependence of dynamic viscosity and fictive strain is presented in Figure 6. In fitting the experimental data, we set η*_∞_ = η*_eq_ from Equation (1). We also fix η*_0_ = η*_inst_, where η*_inst_ was assigned from the first datapoint after the strain down-jump when fitting experimental results and was determined using Equation (3) when predicting behavior outside the range of measured dynamic rheology results. The fitting used four varying parameters, *a*, *s*, *A*, and *β*, for the nonexponential (stretched exponential) version of the model. Only three parameters were allowed to vary for the exponential version of the model, because *β* was fixed at a value of 1 for that case. 

**Figure 7:**
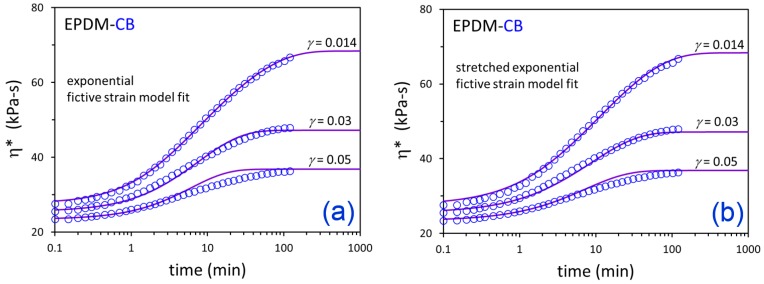
Time-dependent η* data (symbols) after down-jump from equilibrium at *γ* = 0.25 to the indicated *γ* values and fictive strain model fits (solid lines) for EPDM-CB using the exponential (**a**) and stretched exponential (**b**) versions of the model.

**Figure 8:**
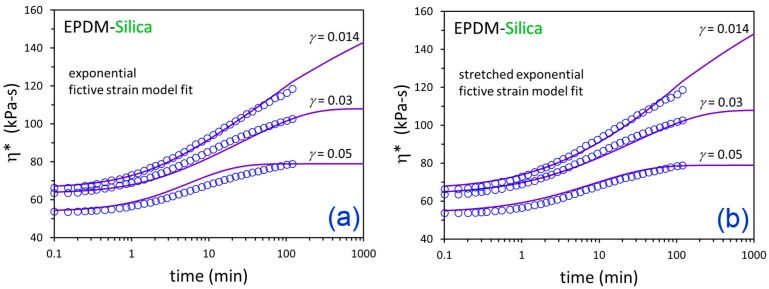
Time-dependent η* data (symbols) after down-jump from equilibrium at *γ* = 0.25 to the indicated *γ* values and fictive strain model fits (solid lines) for EPDM-Silica using the exponential (**a**) and stretched exponential (**b**) versions of the model.

The η*(t) responses from the three flocculation experiments at *γ* = 0.014, 0.03, and 0.05 were simultaneously fit for each material using exponential and nonexponential versions of the fictive strain model. The fits are shown in Figure 7 and Figure 8, and the resulting model parameters are summarized in Table 3. The modeling approach was able to effectively represent the time-dependent dynamic viscosity responses for EPDM-CB and EPDM-Silica systems at three levels of strain down-jump using a single set of fitting parameters for each material. The stretched exponential model gave some modest improvement in fitting compared to the exponential model due to an additional fitting parameter (4 versus 3).

**Figure 9:**
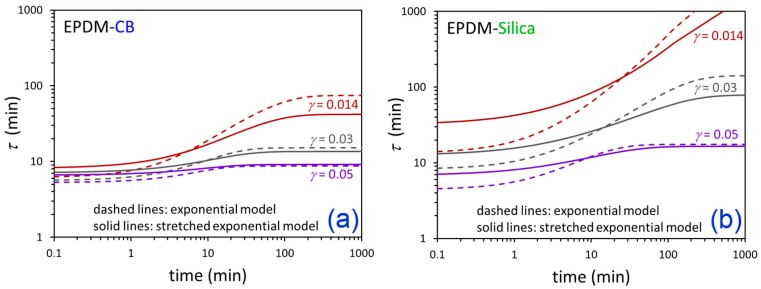
Time-dependent τ from fictive strain model fits for down-jump experiments from equilibrium at *γ* = 0.25 to the indicated *γ* values for EPDM-CB (**a**) and EPDM-Silica (**b**). The dashed lines are from the exponential model and the solid lines are from the stretched exponential model.

The relaxation time using this fictive strain modeling approach is not a constant, nor are there two or more fixed relaxation times. Rather, τ increases with time as the particles become progressively networked/jammed, and the fictive strain is a metric for the evolving filler network structure. Therefore, our methodology describes particle networking/flocculation as a self-limiting process wherein the rate of networking slows down as the filler network builds up. The relaxation time functions from the fitting are compared in Figure 9, where it is evident that untreated silica has more self-limiting filler networking behavior than carbon black in these uncross-linked EPDM nanocomposites. Although the modeling approach does not explicitly include parameters related to filler–filler and polymer–filler interactions, the fitting results show clear differences between the nature of carbon black relative to the significantly more polar silica particles. For both compounds, *s* >> *a*, so the relaxation time depends more on the fictive strain than the actual measurement strain.

**Figure 10:**
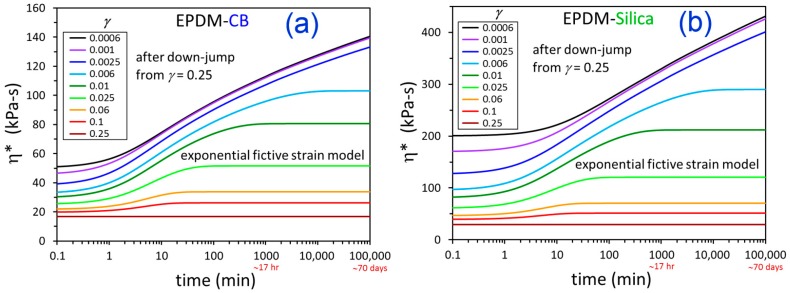
Predicted η* vs. time at the indicated strain amplitudes for EPDM-CB (**a**) and EPDM-Silica (**b**) using parameters from fitting the experimental data (Figure 8 and Figure 9) to the exponential fictive strain model. Note the different y-axis scalings for (**a**,**b**).

The parameters quantified from fitting the experimental data can be used to predict the filler flocculation behavior at *γ* and time conditions that are outside the ranges probed in the dynamic rheological measurements. The results are shown in Figure 10. This exercise showed that filler networking at strain amplitudes below 0.006 (0.6%) is predicted to not reach equilibrium for both EPDM-CB and EPDM-Silica until sometime after a flocculation time of 100,000 min (~70 days), which was the longest time considered in the model predictions. Extending the model to these long-time conditions gives such insights that are not otherwise experimentally possible. It would not be realistic to perform rheological experiments for months, and even mostly saturated EPDM would undergo branching/cross-linking during annealing at 160 °C for this duration. 

**Figure 11:**
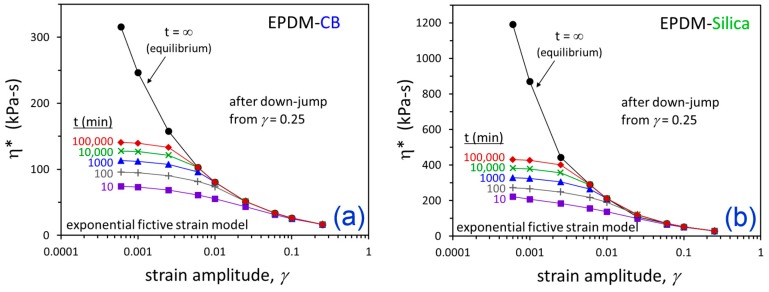
Predicted η* vs. *γ* at the indicated times for EPDM-CB (**a**) and EPDM-Silica (**b**) using parameters from fitting the experimental data (Figure 8 and Figure 9). Note the different y-axis scalings for (**a**,**b**).

**Figure 12:**
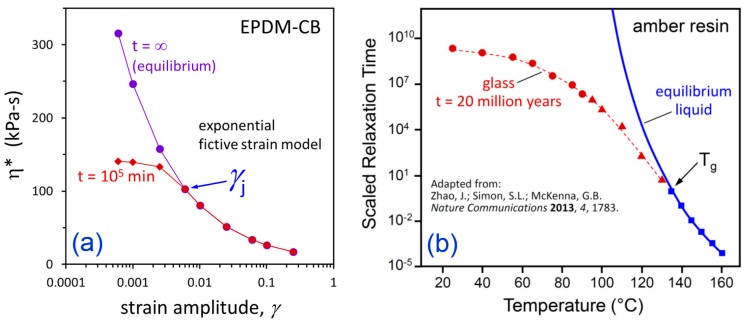
Comparison of departure from equilibrium as strain amplitude is decreased for EPDM-CB (**a**) with departure from equilibrium liquid behavior as temperature is decreased for 20-million-year-old amber resin glass from Zhao, Simon, and McKenna [54] (**b**).

The departure from equilibrium as *γ* is reduced can be viewed by replotting the results in Figure 10 as a function of strain amplitude for various flocculation times in Figure 11. This bears a striking resemblance to the behavior of amorphous polymers and small-molecule glass-formers as they are cooled into the glassy state [53]. Zhao, Simon, and McKenna [54] studied a fossilized amber resin glass and they demonstrated that the glass did not reach the extrapolated equilibrium liquid behavior, even after 20 million years. In Figure 12, we compare our *γ*-dependent predictions for EPDM-CB with their *T*-dependent measurements for the amber resin to further reinforce the known similarities between jammed particle systems and glass-forming materials. A critical jamming transition strain amplitude, *γ_j_*, in particle-filled elastomers is analogous to the glass transition temperature, *T_g_*.

## 4. Conclusions

The fictive strain model was able to capture the time-dependent dynamic viscosity responses for EPDM-CB and EPDM-Silica systems at three levels of strain down-jump using a single set of fitting parameters for each material. Fitting was improved by using a stretched exponential model versus an exponential model, at the expense of introducing an additional fitting parameter (4 versus 3). The utility of this glass-like phenomenological treatment of the time-dependent filler networking/flocculation process in particle-filled elastomers is that it provides new insights into the relative networking characteristics of different polymer nanocomposite systems. Compared to carbon black in filled EPDM compounds, the untreated precipitated silica exhibited more extensive filler networking/flocculation, and the filler network build-up displayed more self-limiting behavior. For both filler types, the relaxation time function was more dependent on structural (fictive) strain than actual strain amplitude, as determined from fitting parameter results indicating *s* >> *a*. 

For dynamic strains less than 0.006 (0.6%), the filler network is predicted to not reach equilibrium, even after 70 days at 160 °C for both filled EPDM materials. There is a tendency to think about the Payne effect in terms of dynamic mechanical properties changing as strain amplitude is increased. However, based on our fictive strain model predictions in Figure 11, we propose that the typical reverse-sigmoidal shape of the Payne effect—as observed for G’ or η* when plotted versus log(*γ*)—is a consequence of the transition from equilibrium state to jammed nonequilibrium filler network as strain amplitude is decreased, similar to the departure of glass-forming liquids into the glassy state as temperature is reduced. A critical strain amplitude for jamming, *γ_j_*, in particle-filled elastomers (polymer nanocomposites) is thus analogous to the glass transition temperature for glass-formers.

The EPDM compounds with silica or carbon black filler were stable during the 155-min-long dynamic rheology experiments at 160 °C, but irreversible dynamic storage modulus increases were noted when the polymer matrix was SBR, presumably due to branching/cross-linking of the unsaturated SBR in the rheometer. We recommend verifying the stability of a material when studying filler flocculation in order to have meaningful interpretation of any model parameters derived from fitting the time-dependent physical phenomenon without any contribution from underlying chemical changes in the polymer–particle system.

## Figures and Tables

**Table 1:** Model formulations for particle-filled rubber compounds (phr).

Compound:	SBR-CB	SBR-Silica	EPDM-CB	EPDM-Silica
SBR	100	100		
EPDM			100	100
Carbon Black (N115)	40		40	
Precipitated Silica		40		40
Antioxidant (6PPD)	2	2	2	2
Total (phr):	142	142	142	142
Filler Volume Fraction, φ:	0.17	0.15	0.16	0.15

**Table 2:** Power Law Parameters for *γ*-dependences of η*_eq_ and η*_inst_.

Compound	η*_eq_ [Equations (1) and (2)]		η*_inst_ [Equation (3)]	
	c (kPa-s)	α	b (kPa-s)	θ
EPDM-CB	8.588	0.486	13.119	0.183
EPDM-Silica	12.535	0.614	19.138	0.317

**Table 3:** Fictive strain model parameters.

Compound	Model	*A* (min)	*a*	*s*	*β*
EPDM-CB	Exponential	3.76	4.13 × 10^−4^	4.14 × 10^−2^	1.0 (fixed)
EPDM-Silica		0.765	9.01 × 10^−3^	1.48 × 10^−1^	1.0 (fixed)
EPDM-CB	Stretched Exponential	5.06	2.47 × 10^−3^	2.71 × 10^−2^	0.835
EPDM-Silica		1.61	2.20 × 10^−2^	9.45 × 10^−2^	0.723
